# Bone Mineral Density in Children and Adolescents with Congenital Adrenal Hyperplasia

**DOI:** 10.1155/2014/806895

**Published:** 2014-03-06

**Authors:** Paulo Alonso Garcia Alves Junior, Daniel Luis Gilban Schueftan, Laura Maria Carvalho de Mendonça, Maria Lucia Fleiuss Farias, Izabel Calland Ricarte Beserra

**Affiliations:** ^1^Universidade Federal do Rio de Janeiro (UFRJ), 21941-901 Rio de Janeiro, RJ, Brazil; ^2^Ambulatório de Endocrinologia, Instituto de Puericultura e Pediatria Martagão Gesteira (IPPMG), UFRJ, Rua Bruno Lobo No. 50, 21941-912 Rio de Janeiro, RJ, Brazil

## Abstract

Chronic glucocorticoid therapy is associated with reduced bone mineral density. In paediatric patients with congenital adrenal hyperplasia, increased levels of androgens could not only counteract this effect, but could also advance bone age, with interference in the evaluation of densitometry. We evaluate bone mineral density in paediatric patients with classic congenital adrenal hyperplasia taking into account chronological and bone ages at the time of the measurement. Patients aged between 5 and 19 years underwent radiography of the hand and wrist followed by total body and lumbar spine densitometry. Chronological and bone ages were used in the scans interpretation. In fourteen patients, mean bone mineral density *Z*-score of total body to bone age was −0.76 and of lumbar spine to bone age was −0.26, lower than those related to chronological age (+0.03 and +0.62, resp.). Mean *Z*-score differences were statistically significant (*P* = 0.004 for total body and *P* = 0.003 for lumbar spine). One patient was classified as having low bone mineral density only when assessed by bone age. We conclude that there was a reduction in the bone mineral density *Z*-score in classic congenital adrenal hyperplasia paediatric patients when bone age was taken into account instead of chronological age.

## 1. Introduction

The risks of osteoporosis and bone fractures are frequently studied in elderly people. It is becoming clear, however, that the amount of bone that is gained during growth is an important determinant of future fracture resistance, since 90% of bone mass is acquired in the first two decades of life [1, 2].

Osteoporosis, which is consequent to low bone mass, deterioration of bone tissue, and disruption of bone architecture, has various origins. Hypogonadism, low calcium intake, vitamin D insufficiency, and the use of certain drugs such as anticonvulsants and glucocorticoids are the main risk factors [3]. The glucocorticoids are considered to be an important component of therapy for several conditions including autoimmune, rheumatic, pulmonary, gastrointestinal, and endocrine disorders [4, 5].

Congenital adrenal hyperplasia (CAH) is an endocrine disorder caused by a deficiency in enzymes responsible for the synthesis of cortisol. With the increase of corticotropin releasing hormone (CRH) and adrenocorticotropic hormone (ACTH), stimulated in response to cortisol deficit, adrenal hyperplasia and overproduction of androgens occur, giving the virilised phenotypic characteristics of the disease. Treatment with corticosteroids increases patient survival, but the optimal dose for the control can be difficult to achieve. High doses may be required, which can compromise bone health [6, 7].

Several studies have examined the status of bone mineral density (BMD) in patients with CAH. Some have shown no difference in the BMD of CAH patients compared with healthy patients, measured by dual-energy X-ray absorptiometry (DXA). Other studies have shown low BMD in all or some subsets of patients with CAH [8–18]. Few studies have evaluated only children [19, 20].

A normal BMD in CAH patients, despite chronic use of glucocorticoids, can be plausible by an androgen excess particular to the disease [7, 21], leading to increased peripheral conversion to oestrogens, opposing the deleterious effect on bone architecture described earlier. Sexual steroids increase osteoblast activity, inhibit the removal of calcium from the body to decrease the formation and activity of osteoclasts, stimulate longitudinal growth of long bones puberty while glucocorticoids promote osteoblast and osteocytes apoptosis, and increase bone resorption by a direct effect on osteoclasts as well as indirect effects that interfere with the metabolism of calcium and vitamin D.

The excessive oestrogen action in paediatric CAH patients causes advanced maturation of the epiphyseal plate, culminating with the usual finding of increased bone age (BA) [22]. We think this could advance the acquisition of peak bone mass compared with healthy children and distort the assessment of DXA, overestimating evaluation of BMD in these patients.

The aim of this study was to evaluate the BMD of total body and lumbar spine by DXA in paediatric patients with CAH classic form, taking into account the chronological age (CA) and BA at the time of the measurement.

## 2. Materials and Methods

Between August 2011 and October 2012, all patients with classic CAH being monitored at the Endocrinology Clinic of Instituto de Puericultura e Pediatria Martagão Gesteira (IPPMG), the paediatric hospital of the Universidade Federal do Rio de Janeiro (UFRJ), were identified and selected to participate in the study. The inclusion criteria were patients with a clinical laboratory diagnosis of classic CAH (salt-wasting or simple-virilising) and age between 5 and 19 years. Exclusion criteria were the presence of other diseases or associated treatment that could interfere with the assessment of BMD in this population including precocious puberty and GnRH analogue use. This study was approved by the Research Ethics Committee of the institute in November 27, 2011, and all parents of patients provided written informed consent to the study.

The medical records of each patient were reviewed to confirm the unequivocal diagnosis of CAH and ascertain associated diseases or treatments. Glucocorticoid therapy in use (converted to hydrocortisone as 10 mg of hydrocortisone is the same of 2 mg of prednisone/prednisolone or 0.375 mg of dexamethasone) [6] was noted. All patients then underwent a complete physical examination by the researcher, including assessment of height, weight, and Tanner puberty stage.

On the date scheduled as routine followup for patients with CAH, radiography of the hand and wrist was performed. BA was evaluated by the researcher and another professional experienced in BA assessment, using as a reference the* Greulich and Pyle* Atlas [23–25]. If there was a discrepancy in the evaluation of the BA, the mean value was used. Difference greater than one year from BA to CA was classified as advanced BA. Close to adult height was considered in patients with BA greater than 15 years in females or greater than 16 in males.

DXA (Lunar Prodigy Advance, GE Healthcare) was performed in a period of up to three months from obtaining the radiographs. All patients underwent the same paediatric protocol preparation, positioning, and image acquisition [26–31]. Patient data (name, birth date, weight, height, and BA) were included in the software (GE Lunar enCORE v.11.40.004). Evaluations of the patients DXA were made by a professional member of the International Society for Clinical Densitometry (ISCD). The results of densitometry were evaluated according to chronological and bone ages. To this end, the program only switches automatically between chronological age and bone age reported to the software, thus assessing bone mass obtained with the aimed age.

BMD assessment comprised total body (less head) densitometry and lumbar spine (L1–L4) densitometry. Data on absolute BMD (g/cm^2^), total body BMD* Z*-score relative to CA (TB* Z* CA), lumbar spine BMD* Z*-score relative to CA (LS* Z* CA), total body BMD* Z*-score relative to BA (TB* Z* BA), and lumbar spine BMD* Z*-score relative to BA (LS* Z* BA) were analysed. Results for BMD* Z*-score less than −2.0 were regarded as low BMD [2].

The database was built with Microsoft Office Excel XP. Statistical analysis was done with XLSTAT software (v. 2012.6.08), including Student's *t-*test for two-paired samples and Pearson correlation for variables investigated, on the basis of a significance level of 5% (*P* < 0.05). Values are shown as mean ± standard deviation.

## 3. Results 

Twenty-two patients were eligible for the study. Fourteen (one male patient) met the inclusion criteria and reached the final stage of the study. Six patients (four male patients) had precocious puberty and were treated with GnRH analogue; two patients (both female) had a history of seizures and anticonvulsant use. As these treatments can interfere with the densitometry analysis, these eight patients were excluded.

The age of the participants ranged from 6.9 to 18.5 years (mean 11.3 ± 3.6 years), and the BA ranged from 5.75 to 19 years (mean 12.9 ± 3.6 years). Nine children had advanced BA (BA − CA ≥ 1) with the largest difference being +4.8 years. Three patients (numbers 3, 12, and 14) were close to their adult height (Table 1).

Four patients (numbers 1, 2, 4, and 7) were at Tanner I stage, with three of these having BA advancement of at least two years. Regarding the use of glucocorticoids, two were being treated with hydrocortisone, eight with prednisolone, three with prednisone, and one with dexamethasone. All patients started glucocorticoid treatment before 4 months of life. The mean hydrocortisone equivalent dose was 12.9 mg/m^2^ per day. Eight patients had salt-wasting form and received fludrocortisone at a dose of 0.1 mg/day (Table 2).

Regarding the assessment of BMD, Table 3 shows the data obtained from the total body and lumbar spine DXA of the 14 patients.

Table 3 also shows the conversion of total body and lumbar spine BMD to BMD* Z*-score to CA and BA. The conversion of these values using the software database for healthy patients of the same CA gave a mean TB BMD* Z*-score of 0.03 ± 1.09 with a minimum and maximum value of −1.2 and +3.5, respectively, whereas the LS BMD* Z*-score average was 0.62 ± 1.11, with a lower limit of −1.0 and upper limit of +3.2. The analysis of these values, taking into account this time BA, gave a mean TB BMD* Z*-score of −0.76 ± 0.82 with minimum value of −1.9 and maximum value of +0.5. In LS BMD* Z*-score to BA; the mean values were −0.26 ± 1.11 ranging from −2.1 to +1.5.

All nine patients with advanced BA had lower values when BMD* Z*-score to BA was compared with BMD* Z*-score to CA. For total body, all patients had normal BMD* Z*-score when measured to CA and BA. For lumbar spine, all patients had normal BMD* Z*-score to CA, with one (patient 2) having low BMD to BA.

**Figure 1:**
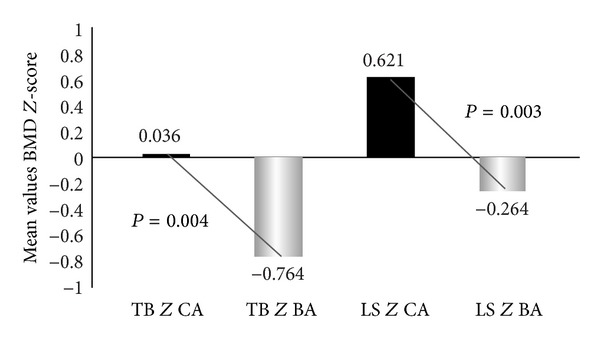
Total body and lumbar spine mean BMD* Z*-scores to chronological and bone ages. TB* Z* CA: total body BMD* Z*-score to CA, TB* Z* CA: total body BMD* Z*-score to BA. LS* Z* CA: lumbar spine BMD* Z*-score to CA, LS* Z* BA: lumbar spine BMD* Z*-score to BA.

According to the Student's *t*-test for two-paired samples, the differences between the mean values of TB* Z* CA versus TB* Z* BA and of LS* Z* CA versus LS* Z* BA were statistically significant, with lower values for BA (*P*  0.004 and *P*  0.003, resp.) (Figure 1).

Using the Student's *t*-test and Pearson correlation, we found moderate statistically significant correlation between TB* Z* CA and height* Z*-score. Moderate correlation, but not significant, between height* Z*-score and TB* Z* BA, between hydrocortisone dose in use and BMD* Z*-scores assessed, and between BA − CA difference and BMD* Z*-scores assessed. Other correlations were assessed, but they were not significant as described in Table 4.

## 4. Discussion

The studies evaluating BMD in patients with CAH are important since corticosteroid therapy is crucial in this type of disease. Reisch et al. [17] carried out an extensive review of bone health in patients with CAH and observed discrepant findings for BMD that ranged from normal to low. Two studies have included only children: Elnecave et al. [20] and de Almeida Freire et al. [19] found normal values of BMD* Z*-score to CA. de Almeida Freireet al. also assessed values of BMD* Z*-score to BA, finding lower values than those evaluated for CA. As this was, however, not the focus of their study, it was not discussed in detail.

In CAH, an increase in androgens circulation and their subsequent conversion into oestrogens influences bone maturation and closure of the growth plate in children and adolescents. This is easily verified by the detection of BA advancement, which is a common finding in CAH patients [32, 33]. The discrepancy between CA and BA can interfere with BMD measurement by DXA, since this examination, in the standard mode, only takes into account the CA of patients for analysis. Thus, the BMD of the patients should not be compared with that of a healthy population with the same CA as is usually done for evaluation of children DXA data.

One way to avoid this confounding factor in patients with advanced bone maturation would be the evaluation of the BMD with the BA. The inclusion of this analysis is done by simply adding the BA value into the software for the densitometer. This does not increase the cost and does not require an additional scan.

It should be noted that longer half-life glucocorticoids such as prednisone and prednisolone were commonly administered to our CAH patients as steroid replacement due to lack of availability of stable hydrocortisone oral formulation in our country. Leiteet al. [34] found no difference in the long-term use of these glucocorticoids by CAH patients.

On analysing the individual BMD data in our study, all patients evaluated by CA were classified as having normal density. The same occurred with the analysis of total body BMD to BA, but when it was assessed by lumbar spine, the LS* Z* BA of one patient (number 2) was less than −2, classified as having low BMD [24].

Moderate correlation, statistically significant, between height* Z*-score and TB* Z* CA, was found in our study. Other correlations evaluated were not significant, probably because the small number of participants, but we clearly demonstrated a reduction in BMD* Z*-score, both total body and lumbar spine, when we took into account BA instead of CA (mean BMD* Z*-score to BA was statistically lower than the mean BMD* Z*-score to CA).

Interestingly, only three of the 14 patients had reached almost adult height, giving a possible potential for worsening of these BMD* Z*-scores in the patients with growth outlook with maintenance of current glucocorticoid treatment in the next few years.

The limitation of our study was the small number of participants limited to paediatric CAH classic form without use of GnRH analogue. We believe that these careful selection criteria have been a differential in the international literature and may stimulate research centers with more numbers of patients in using BA in DXA perform.

Unfortunately we could not assess the DXA according to Tanner stage of the participants because the limited number of patients included. Note that the evaluation of BMD stratified by stage of puberty finds several questions in the international literature as well as few comparative data. This assessment in CAH patients is even more difficult. The early adrenarche without necessarily developing central precocious puberty could classify patients with advanced pubertal stages. We believe that when comparing BMD with bone age, which has relationship with pubertal stage, we minimize errors of pubertal compared.

## 5. Conclusions

There was a reduction in the BMD* Z*-score in paediatric patients with classic CAH when BA was taken into account rather than CA. These results cause us concern about the real risk, only now viewed with the use of BA assessment by DXA methodology, of bone fractures and osteoporosis later in life in patients diagnosed with classic CAH. We have thus shown the importance of using the degree of maturation (BA) of children and adolescents for an accurate assessment of BMD by DXA in this subgroup of patients.

## Figures and Tables

**Table 1:** Patient characteristics according togender, chronological age, bone age, difference between bone age and chronological age, and *Z*-score for height.

Patient	Gender	CA (years)	BA (years)	ΔBA − CA	*Z* height
1	F	6.9	5.75	–1.15	–0.29
2	F	7.4	11	3.6	–0.07
3	F	18.5	19	0.5	–0.47
4	F	7.5	10	2.5	0.16
5	F	9.2	14	4.8	1.88
6	M	15.4	15	−0.4	–1.34
7	F	8.1	11	2.9	–1.07
8	F	12.4	14	1.6	–0.25
9	F	12	14	2	–0.95
10	F	11.3	11	–0.3	–0.18
11	F	8.5	11	2.5	1.65
12	F	15.8	19	3.2	–4.11
13	F	10.9	10	–0.9	0.12
14	F	14.8	16	1.2	–2.13

Mean ± SD	—	11.3 ± 3.6	12.9 ± 3.6	1.57 ± 1.81	−0.50 ± 1.47

F: female, M: male, CA: chronological age, BA: bone age, *Z* height: height *Z*-score, and SD: standard deviation.

**Table 2:** Patient characteristics according to gender, congenital adrenal hyperplasia form, Tanner stage, type of corticosteroid treatment, hydrocortisone dose equivalent, and use of fludrocortisone.

Patient	Gender	CAH form	Tanner stage	Corticosteroid treatment	Hydrocortisone dose (mg/m^2^/day)	Fludrocortisone dose (mg/day)
1	F	S.W.	M1P1	Prednisolone	4	Y (0.1)
2	F	S.W.	M1P1	Hydrocortisone	18	Y (0.1)
3	F	S.W.	M4P4	Prednisone	6	Y (0.1)
4	F	S.W.	M1P1	Hydrocortisone	15	Y (0.1)
5	F	S.W.	G4P4	Prednisolone	24	Y (0.1)
6	M	S.W.	M5P5	Dexamethasone	3.5	Y (0.1)
7	F	S.V.	M1P1	Prednisolone	14.8	N
8	F	S.V.	M2P2	Prednisolone	11.2	N
9	F	S.V.	M4P5	Prednisolone	18.8	N
10	F	S.W.	M2P4	Prednisolone	8	Y (0.1)
11	F	S.V.	M2P4	Prednisone	18	N
12	F	S.V.	M1P5	Prednisone	14.9	N
13	F	S.V.	M2P1	Prednisolone	3.6	N
14	F	S.W.	M5P5	Prednisolone	11.6	Y (0.1)

CAH: congenital adrenal hyperplasia, F: female, M: male, S.V.: simple-virilising, S.W.: salt-wasting, Y: yes, and N: no.

**Table 3:** Total body and lumbar spine BMD absolute values and conversion for *Z*-score to chronological and bone ages.

Patient	TB BMD	LS BMD	TB *Z* CA	TB *Z* BA	LS *Z* CA	LS *Z* BA
1	0.808	0.737	−0.3	0.5	0.8	1.5
2	0.849	0.648	0.4	−1.3	−0.3	−2.1
3	1.167	1.211	−0.5	−0.5	−0.2	−0.2
4	0.870	0.811	0.7	−0.5	1.5	−0.1
5	1.088	1.074	3.5	0.3	3.2	0.2
6	1.105	1.204	0.0	0.0	0.6	0.6
7	0.855	0.744	−0.5	−1.6	0.2	−1.4
8	1.020	1.190	−0.2	−1.1	1.7	0.5
9	0.936	1.001	−0.4	−1.3	0.6	−0.5
10	0.955	1.041	0.1	0.1	1.3	1.3
11	0.800	0.706	−0.6	−1.8	0.1	−1.5
12	1.125	1.339	−0.4	−1.5	1.1	0.5
13	0.912	0.727	−0.1	−0.1	−1.0	−1.0
14	1.028	0.998	−1.2	−1.9	−0.9	−1.5

Mean (±SD)			0.03 (±1.09)	−0.76 (±0.82)	0.62 (±1.11)	−0.26 (±1.11)

TB BMD: total body BMD absolute values, LS BMD: lumbar spine BMD absolute values, TB *Z* CA: total body BMD *Z*-score to CA, TB *Z* BA: total body BMD *Z*-score to BA, LS *Z* CA: lumbar spine BMD *Z*-score to CA, LS *Z* BA: total body BMD *Z*-score to BA, and SD: standard deviation.

**Table 4:** Pearson correlation between height *Z*-score, hydrocortisone dose and BA less CA, and BMD *Z*-scores with *P* value.

	TB *Z* CA	TB *Z* BA	LS *Z* CA	LS *Z* BA
Height *Z*-score	0,53 (0,046)	0,35 (0,208)	0,26 (0,357)	−0,10 (0,708)
Hydrocortisone dose	0,49 (0,086)	−0,48 (0,091)	0,44 (0,130)	−0,42 (0,145)
ΔBA − CA	0,48 (0,079)	−0,45 (0,099)	0,40 (0,148)	−0,41 (0,144)

TB *Z* CA: total body BMD *Z*-score to CA, TB *Z* BA: total body BMD *Z*-score to BA, LS *Z* CA: lumbar spine BMD *Z*-score to CA, LS *Z* BA: total body BMD *Z*-score to BA, BA: bone age, and CA: chronological age.

## Abbreviations

BA: Bone age
BMD: Bone mineral density
CA: Chronological age
CAH: Congenital adrenal hyperplasia
DXA: Dual-energy X-ray absorptiometry
ISCD: International Society for Clinical Densitometry
LS* Z* BA: Lumbar spine BMD* Z*-score relative to bone age
LS* Z* CA: Lumbar spine BMD* Z*-score relative to chronological age
TB* Z* BA: Total body BMD* Z*-score relative to bone age
TB* Z* CA: Total body BMD* Z*-score relative to chronological age.
